# Identifying key genes and functionally enriched pathways in Th2-high asthma by weighted gene co-expression network analysis

**DOI:** 10.1186/s12920-022-01241-9

**Published:** 2022-05-12

**Authors:** Yao Cao, Yi Wu, Li Lin, Lin Yang, Xin Peng, Lina Chen

**Affiliations:** 1 Division of Pediatric Pulmonology and Immunology, West China Second University Hospital, Sichuan University, Chengdu, Sichuan People’s Republic of China; 2 Key Laboratory of Birth Defects and Related Diseases of Women and Children (Sichuan University), Ministry of Education, Chengdu, Sichuan People’s Republic of China; 3 NHC Key Laboratory of Chronobiology (Sichuan University), Chengdu, People’s Republic of China

**Keywords:** Asthma, Weighted gene coexpression network analysis (WGCNA), Hub gene, Gene ontology

## Abstract

**Background:**

Asthma is a chronic lung disease characterized by reversible inflammation of the airways. The imbalance of Th1/Th2 cells plays a significant role in the mechanisms of asthma. The aim of this study was to identify asthma-related key genes and functionally enriched pathways in a Th2-high group by using weighted gene coexpression network analysis (WGCNA).

**Methods:**

The gene expression profiles of GSE4302, which included 42 asthma patients and 28 controls, were selected from the Gene Expression Omnibus (GEO). A gene network was constructed, and genes were classified into different modules using WGCNA. Gene ontology (GO) was performed to further explore the potential function of the genes in the most related module. In addition, the expression profile and diagnostic capacity (ROC curve) of hub genes of interest were verified by dataset GSE67472.

**Results:**

In dataset GSE4302, subjects with asthma were divided into Th2-high and Th2-low groups according to the expression of the SERPINB2, POSTN and CLCA1 genes. A weighted gene coexpression network was constructed, and genes were classified into 7 modules. Among them, the red module was most closely associated with Th2-high asthma, which contained 60 genes. These genes were significantly enriched in different biological processes and molecular functions. A total of 8 hub genes (TPSB2, CPA3, ITLN1, CST1, SERPINB10, CEACAM5, CHD26 and P2RY14) were identified, and the expression levels of these genes (except TPSB2) were confirmed in dataset GSE67472. ROC curve analysis validated that the expression of these 8 genes exhibited excellent diagnostic efficiency for Th2-high asthma and Th2-low asthma.

**Conclusions:**

The study provides a novel perspective on Th2-high asthma by WGCNA, and the hub genes and potential pathways involved may be beneficial for the diagnosis and management of Th2-high asthma.

**Supplementary Information:**

The online version contains supplementary material available at 10.1186/s12920-022-01241-9.

## Background

Asthma is a common chronic inflammatory disease of the lower airway characterized by airflow obstruction, bronchial hyperresponsiveness, and airway inflammation [1]. The CD4 T-helper cell type 2 (Th2)-mediated pathway in the airway epithelium is critical in allergic asthma [2]. Drugs targeting Th2 cytokines (IL-4, IL-5, and IL-13) have been developed and shown efficacy in recent studies [3, 4]. However, the etiology and progression of asthma are still under investigation.

Although multiple studies have shown differences in cytokine expression between Th2-high and Th2-low asthma [4, 5], it is not known whether the gene signature of bronchial airway epithelium can identify Th2-high and Th2-low endotypes. Weighted gene coexpression network analysis (WGCNA) is a useful tool to identify functional pathways and candidate biomarkers and has recently been used to explore the complex relationships between gene network signatures and complicated phenotypes [6]. Meanwhile, WGCNA has been successfully adopted to investigate the gene-network signature, coexpression modules, and hub genes in respiratory diseases, such as severe asthma [7–9] and chronic obstructive pulmonary disease (COPD) [10–12].

In this study, we analyzed gene expression in asthmatic patients and healthy control subjects and measured the expression of genes related to type 2 inflammation. We aimed to identify hub genes through WGCNA to further explore the potential mechanism underlying Th2-high asthma.

## Materials and methods

### Microarray data collection

We downloaded mRNA expression profiles GSE4302 and GSE67472 from the Gene Expression Omnibus (GEO, https://www.ncbi.nlm.nih.gov/geo). Specifically, airway epithelial brushings for microarray analysis of 42 subjects with asthma (not on inhaled steroids) and 28 healthy controls were obtained from GSE4302. Airway epithelial brushings for microarray analysis in GSE67472 were obtained from 62 subjects with mild-to-moderate asthma (not on inhaled steroids) and 43 healthy controls. Asthma subjects in the GSE4302 microarray dataset were divided into Th2-high and Th2-low asthma based on their expression of a three-gene signature of type 2 inflammation: SERPINB2, POSTN and CLCA1 [2, 12].

### WGCNA

The coefficient of variation (CV) of each gene in the GSE4302 microarray dataset was calculated, and genes with CV > 5% (4133 genes) were used to construct a gene coexpression network using the WGCNA package (version 1.69) [6] in R. Firstly, the outlier samples were removed by hierarchical cluster analysis. Secondly, we constructed a scale-free network (*R*^2^ = 0.9) based on the criteria that soft-thresholding power β was set as 10 using the pickSoftThreshold function. Subsequently, the adjacency gene network was transformed into a topological overlap matrix (TOM), and the corresponding dissimilarity (1-TOM) was calculated. Lastly, dynamicTreeCut algorithm was used to identify modules, and 30 was chosen as the minimum number of genes in each module. A value of 0.25 was defined as the threshold for cut height to merge possible similar modules.

### Identification of clinical significant modules

To further analyze the module, module eigengenes (ME) was calculated, which is the principal component of each gene module and could be considered as a representative of all genes in a given module. The correlation between genes and ME was defined as module membership (MM), and the correlation between the genes and clinical trait was defined as the gene significance (GS). The ME values were correlated with control, Th2-high and Th2-low groups by Pearson's correlation. Finally, the module highly correlated with clinical traits of Th2-high asthma was selected for further analysis.

### Functional enrichment analysis

To obtain further insights into the function of the genes in the module most related to Th2-high asthma, Gene Ontology (GO) analysis was performed and visualized with the R package “Clusterprofiler” (version 3.18.0) [13]. The ontology contains three categories: molecular function (MF), biological process (BP), and cellular component (CC). A Q value < 0.05 was set as the cutoff.

### Identification and validation of hub genes

In the module-trait correlation analysis, genes with absolute GS greater than 0.4 and high within-module connectivity of the modules (absolute MM > 0.8) were considered hub genes. The hub genes were further validated in datasets GSE67472. We screened the differentially expressed genes (DEGs) from Th2-high asthma vs. Th2-low asthma and Th2-high asthma vs. healthy controls using the “limma” R package. DEGs were considered genes with a false discovery rate (FDR) < 0.05 and absolute log2 fold change (FC) ≥ 1. Among the hub genes, eight genes (TPSB2, CPA3, ITLN1, CST1, SERPINB10, CEACAM5, CHD26, P2RY14) were further validated in another dataset GSE67472. The area under the receiver operating characteristics (ROC) curve (AUC) was calculated, and an ROC curve was plotted with the “ROCR” package to evaluate the capability of hub genes to distinguish Th2-high asthma and Th2-low asthma.

## Results

### Asthma phenotyping based on epithelial markers

**Fig. 1:**
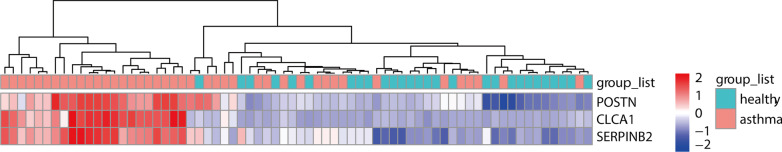
Heatmap showing unsupervised hierarchical clustering of POSTN, CLCA1, and SERPINB2 expression levels in bronchial epithelium. Red represents a high expression level, and green represents a low expression level

Seventy subjects (28 healthy control subjects and 42 asthma subjects) in datasets GSE4302 underwent unsupervised hierarchical clustering based on the microarray expression levels of SERPINB2, POSTN and CLCA1 (Fig. 1). Approximately half of the asthma subjects (n = 22) with consistently high expression of a signature of Type 2 inflammation (SERPINB2, POSTN and CLCA1) were identified as Th2-high asthma, and 20 asthma subjects were identified as Th2-low asthma.

### Weighted coexpression network construction

**Fig. 2:**
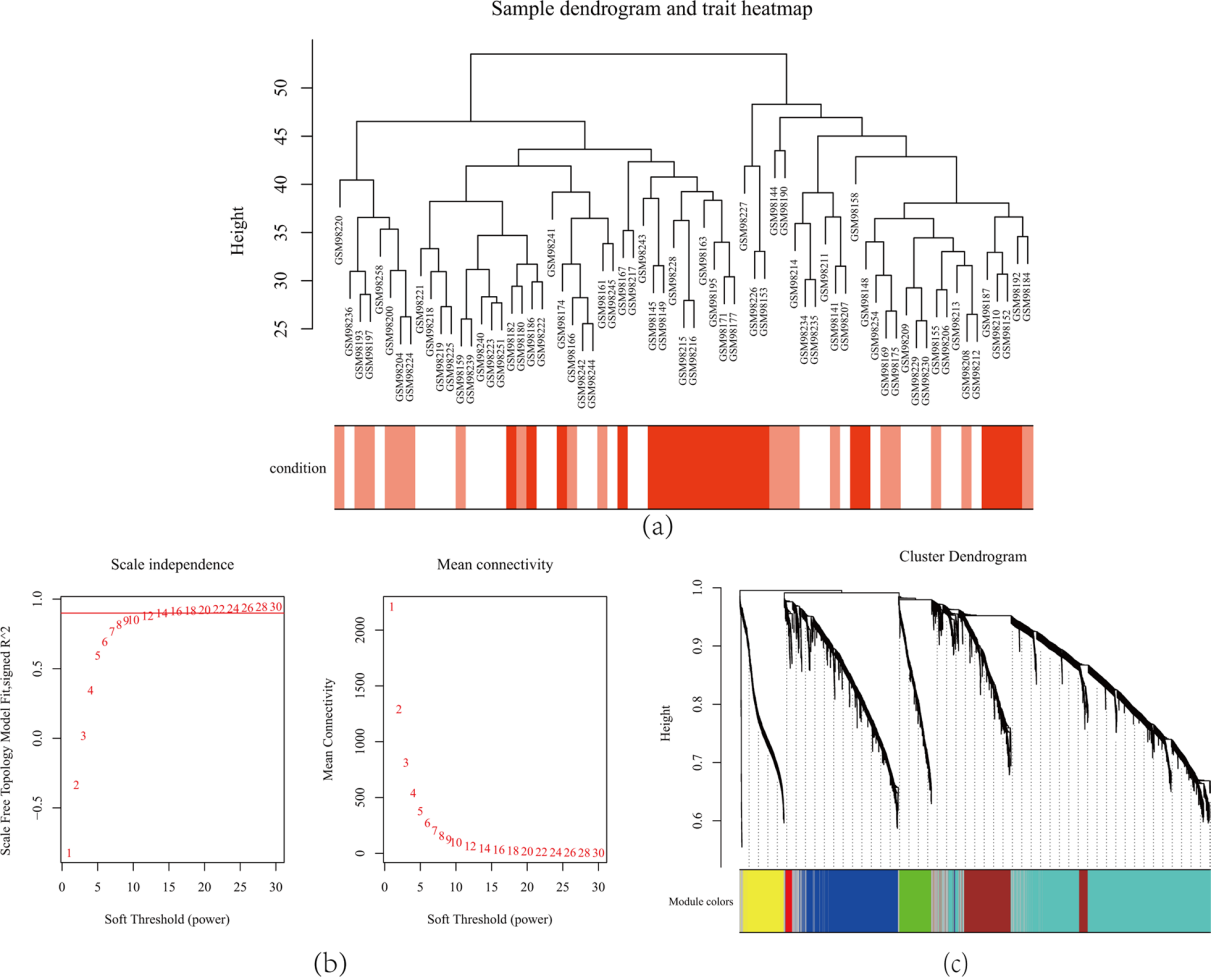
**a** Clustering dendrogram of 69 samples. **b** Determination of soft-thresholding power in the WGCNA. Analysis of the scale-free fit index for various soft-thresholding powers (β) and analysis of the mean connectivity for various soft-thresholding powers. **c** Clustering dendrogram of differentially expressed genes based on a dissimilarity measure (1-TOM)

The GSE4302 samples were clustered using hierarchical agglomerative clustering with average linkage, and one outlier sample (GSM98201) was removed (Fig. 2a). In this study, GSE4302 was used to construct coexpression networks. The power of β = 10 (scale-free R^2^ = 0.9) was selected as the correlation coefficient threshold to ensure a scale-free network (Fig. 2b). A total of seven modules were identified through WGCNA (Fig. 2c).

### Key modules and hub genes identification

**Fig. 3:**
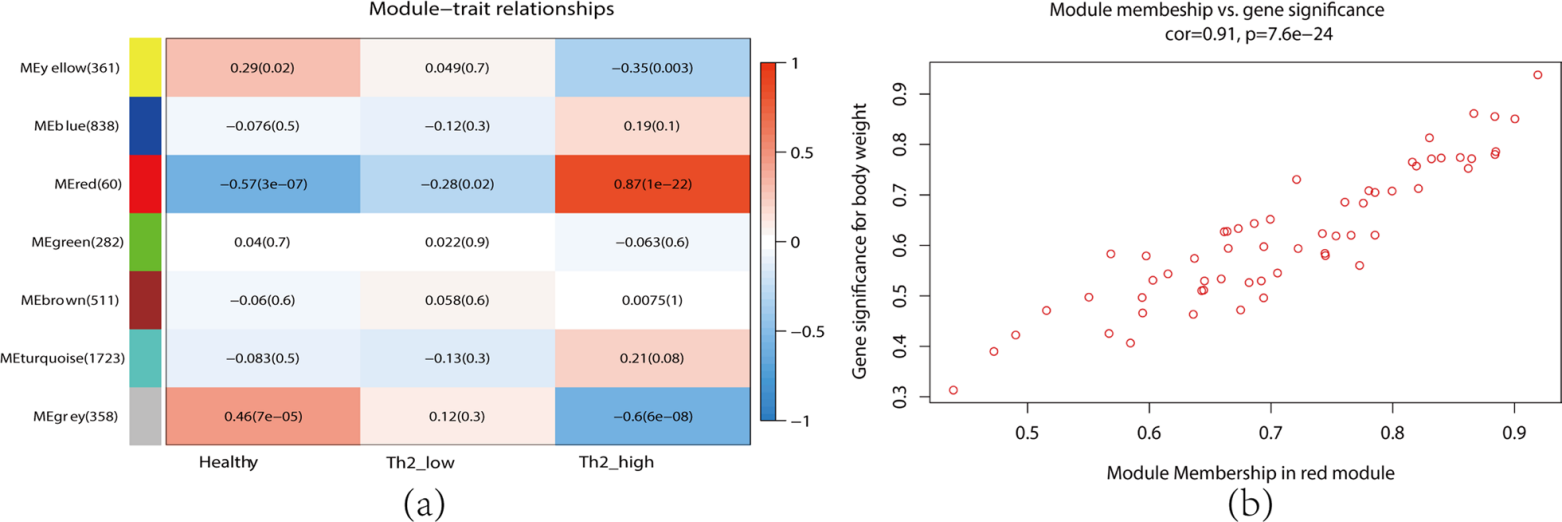
**a** Heatmap of the correlation between module and clinical traits (each cell contained the correlation coefficient and corresponding P value). **b** The gene significance for Th2-high asthma in the red module (one dot represents one gene in the red module)

Module-trait relationship analyses showed that multiple modules were related to Th2-high asthma, and the red module, which included 60 genes, was most significantly associated with Th2-high asthma (Fig. 3a). This module was identified as the clinically significant module for further analysis. Figure 3b shows the significance of these genes in the red module, and 15 genes with high connectivity in the clinically significant module were identified as hub genes based on the cutoff criteria (|GS|> 0.4 and |MM|> 0.8). The detailed gene information of each module was listed in Additional file 1.

### Gene ontology of the key co-expression module

**Fig. 4:**
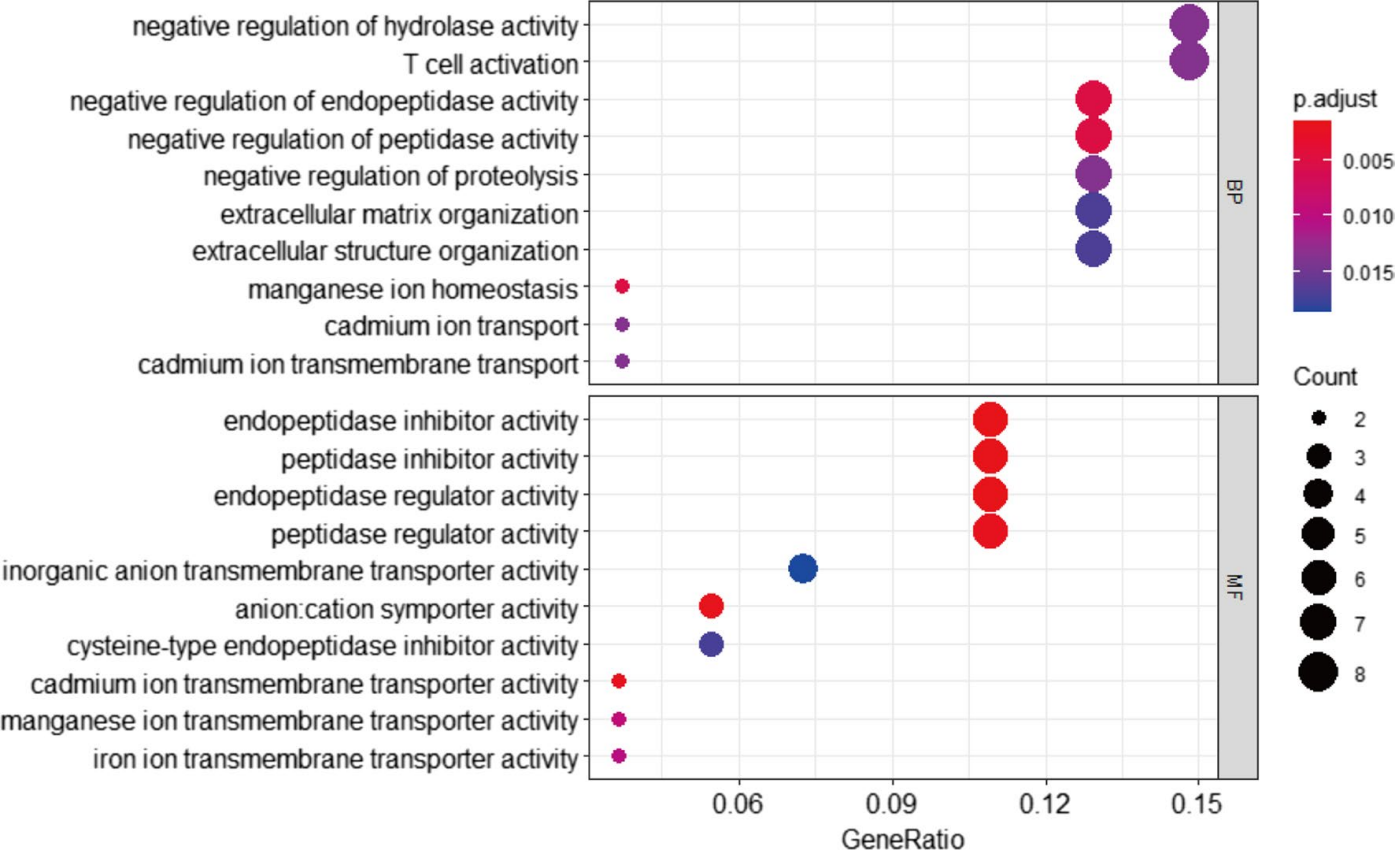
Gene ontology of red module genes including biological process (BP) analysis and molecular function (MF) analysis

GO functional enrichment analysis of the genes in the clinically significant module showed that the genes were mainly enriched in BP and were involved in negative regulation of hydrolase activity, T cell activation and negative regulation of peptidase and endopeptidase activity (Fig. 4). The genes in the MF category were mainly enriched in endopeptidase/peptidase inhibitor activity and endopeptidase/peptidase regulator activity (Fig. 4). However, the genes in the CC group showed no enrichment results. These results indicated that these genes could be related to multiple biological pathways orchestrating Th2-high asthma development.

### Validation of hub genes

**Fig. 5:**
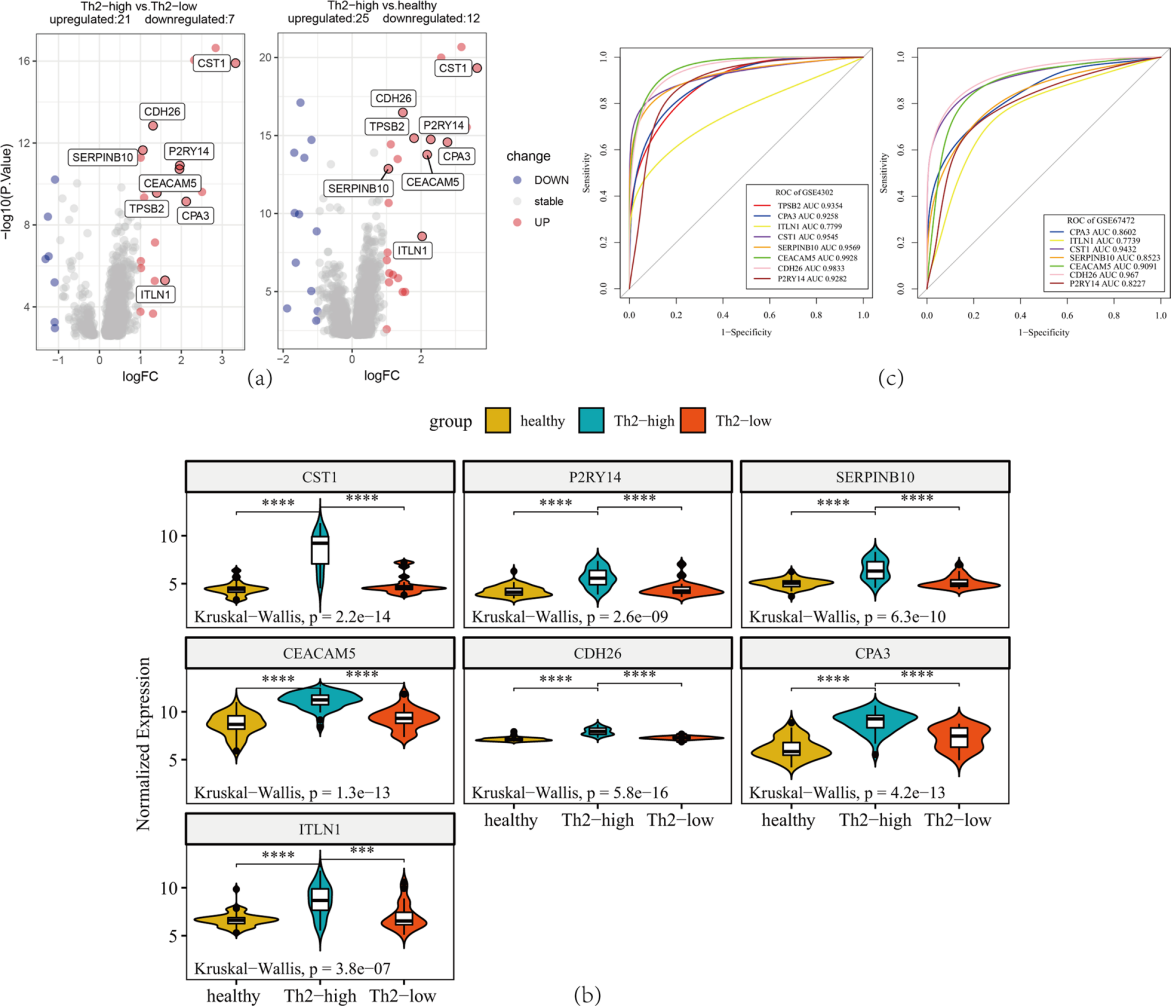
Validation of hub genes in datasets GSE4302 and GSE67472. **a** Volcano plot for differential gene expression in dataset GSE4302. **b** The expression levels of CST1, P2RY14, SERPINB10, CEACAM5, CDH26, CPA3 and ITLN1 were significantly upregulated in Th2-high asthma patients in dataset GSE67472. The Kruskal–Wallis test was used to evaluate the statistical significance of differences. **c** ROC curve of hub genes in dataset GSE4302 and dataset GSE67472

In dataset GSE4302, 27 DEGs were identified in the group of Th2-high asthma vs. Th2-low asthma (Fig. 5a), and 38 DEGs were identified in the Th2-high asthma vs. healthy group (Fig. 5a). The expression of eight hub genes (CST1, P2RY14, SERPINB10, CEACAM5, CDH26, CPA3, TPSB2, and ITLN1) was significantly upregulated in Th2-high asthmatic subjects compared to Th2-low asthmatic subjects and healthy subjects (Fig. 5a). Moreover, the expression levels of the seven hub genes were investigated in another dataset, GSE67472 (the gene TPSB2 expression data were not found in GSE67472). The seven genes were also significantly upregulated in the airway epithelium of Th2-high asthma patients compared to Th2-low asthma patients and controls in dataset GSE67472 (Fig. 5b). In addition, the ROC curve indicated that these hub genes exhibited excellent diagnostic efficiency for Th2-high asthma and Th2-low asthma in datasets GSE4302 and GSE67472 (Fig. 5c).

## Discussion

Bronchial asthma a highly heterogenous disease. Based on the characteristics of inflammation, bronchial asthma can be identified into two endotypes, type 2 asthma and nontype 2 asthma [14–16]. Th2 cells and cytokines (an imbalance of Th1/Th2) play a major role in type 2 inflammation, which is considered a major pathophysiological mechanism of asthma [2, 4]. Several reports have identified a typical Th2 feature characterized by elevated Th2-type inflammation in the lower airway [7, 17]. In another study, gene expression in patients with mild asthma and healthy control subjects found that Th2 signature genes in lower airway epithelial cells were associated with atopy and eosinophilic airway inflammation [18, 19].

There is growing interest in seeking biomarkers to distinguish Th2-high and Th2-low phenotypes and to predict responsiveness to treatment [20]. At present, several biomarkers, such as fractional exhaled nitric oxide (FeNO), serum IgE, blood or sputum eosinophils and serum periostin, have been adopted [21]. Bhakta et al. reported that the three-gene mean of periostin, CLCA1 and serpinB2 in airway epithelial brushings identifies Th2-high and Th2-low populations and predicts FEV1 improvement with inhaled corticosteroid (ICS) [22].

WGCNA can provide a deep understanding of gene networks, hub genes and pathogenesis associated with asthma. In this study, we speculated that assessing gene expression in airway epithelial cells using WGCNA may identify the gene-network signature, provide potential biomarkers of Th2-high asthma and further predict treatment responses.

Seven modules were identified as significantly correlated with asthma status. The red module observed in this study was positively correlated with asthma traits and regarded as the most important module in Th2-high asthma pathogenesis. It was further analyzed using a GO enrichment method and the results showed it to be mainly enriched in endopeptidase and peptidase inhibitor/regulator activity. Then, key genes of TPSB2, CPA3, ITLN1, CST1, SERPINB10, CEACAM5, CHD26, and P2RY14 were identified.

The above eight hub genes were validated in another dataset GSE67472. We found that their expression levels (except TPSB2, which had not been studied in dataset GSE67472) were also significantly upregulated in Th2-high asthma patients compared to controls in dataset GSE67472. The diagnostic efficiencies of hub genes were evaluated by ROC curve analysis. Notably, the AUC of ROC curves were all more than 0.750 for the eight hub genes, indicating a high diagnostic value. This suggested that these hub genes probably participate in the pathogenesis and immune imbalance and could be used as potential biomarkers for the diagnosis and therapeutic targets of Th2-high asthma.

Although the mechanisms underlying asthma are unclear, some of these hub genes are under investigation [23–29]. For example, CST1 is a member of the type 2 cystatin (CST) superfamily, which is known to inhibit the proteolytic activities of cysteine proteases. Increased CPA3 expression in intraepithelial mast cells among Th2-high asthma patients was observed [23]. Yan et al. discussed the coexpressed DEGs of CST1 and CPA3 and found that there were significant correlations of the CST1 and CPA3 genes with novel biomarkers involved in the comorbidity of rhinitis and asthma [24]. In addition, Natasha A Winter et al. found that the gene expression of TPSAB1/TPSB2 and CPA3 correlated with sputum mast cells/basophils, and in severe asthma, TPSB2 and CPA3 were associated with eosinophilic airway inflammation and blood eosinophils [25].

The hub gene ITLN1, which is expressed in airway epithelial cells and involved in inflammatory pathways downstream of IL-13, was found to be significantly upregulated in the bronchial brushings of patients with asthma compared with controls [26, 27]. It has been reported that ITLN1 is a biomarker associated with disease susceptibility in asthma [28]. A study on ERPINB10-knockdown mice found that these groups of mice had diminished numbers of Th2 cells and were more susceptible to apoptosis, indicating that SERPINB10 may contribute to allergic inflammation and the Th2 response of asthma by inhibiting the apoptosis of Th2 cells [29].

In general, our study identified some hub genes through WGCNA to further explore the potential mechanism underlying Th2-high asthma. This may provide a better molecular characterization of asthma and consequent establishment of new pharmacological targets. Furthermore, the Th2-high subgroup of asthma is corticosteroid sensitive and benefits from biologic agents [4, 30]. In this respect, future research on the function of these genes not only helps promote our understanding of Th2-high and Th2-low endotypes of asthma but may also help to guide treatment strategies and predict response to treatment.

However, there were some limitations to our study. On one hand, the study only presented data mining and data analysis, and did not perform experiments to further study the exact mechanism of the identified hub genes in asthma. On the other hand, the analysis was based on limited genetic data with small sample sizes. Thus, the expression of these identified hub genes still needs to be validated in more datasets, and the exact mechanisms also need more exploration.

## Conclusion

In summary, our study performed WGCNA to provide a framework of coexpression gene modules of Th2-high asthma and led to the identification of some hub genes that may help us to better understand the underlying pathogenesis and guide treatment decisions for the Th2-high subgroup of asthma. The exact molecular mechanisms of the hub genes and functional pathways in Th2 asthma still need to be further explored.

## Supplementary Information

**Additional file 1**. The detailed gene information of each module.

## Data Availability

Our data can be found in the Gene Expression Omnibus (GEO, https://www.ncbi.nlm.nih.gov/geo/, GSE4302 and GSE67472) database.

## Abbreviations

WGCNA: Weighted gene coexpression network analysis
GEO: Gene Expression Omnibus
GO: Gene ontology
Th2: T-helper cell type 2
COPD: Chronic obstructive pulmonary disease
CV: Coefficient of variation
MF: Molecular function
BP: Biological process
CC: Cellular component
DEGs: Differentially expressed genes
FDR: False discovery rate
FC: Fold change
FeNO: Fractional exhaled nitric oxide
ICS: Inhaled corticosteroid
